# Assessment of the Penetration of an Endodontic Sealer into Dentinal Tubules with Three Different Compaction Techniques Using Confocal Laser Scanning Microscopy

**DOI:** 10.3390/jfb14110542

**Published:** 2023-11-07

**Authors:** Ignacio Barbero-Navarro, Diego Velázquez-González, María Esther Irigoyen-Camacho, Marco Antonio Zepeda-Zepeda, Paulo Mauricio, David Ribas-Perez, Antonio Castano-Seiquer

**Affiliations:** 1 Dental School, University Institute Egas Moniz (IUEM), 2800-064 Almada, Portugal; ibarbero2@us.es (I.B.-N.);; 2 Dental School, University of Seville, 41009 Seville, Spain; acastano@us.es; 3 Health Care Department, Autonomous Metropolitan University-Xochimilco, Mexico City 04960, Mexico; mzepeda@correo.xoc.uam.mx; 4 Interdisciplinary Research Centre, University Institute Egas Moniz (IUEM), 2800-064 Almada, Portugal

**Keywords:** endodontic obturation, obturation technique, sealers’ penetration depth, dentin tubule penetration, confocal laser scanning microscope

## Abstract

Adequate root canal sealing is essential for the success of endodontic treatment. There are numerous techniques available; identifying simple and efficient techniques is important to provide good patient care. The purpose of the study was to compare the maximum penetration depth and the percentage of sealant penetration of an endodontic sealer into dentine tubules using cold lateral condensation, continuous wave, and hybrid techniques, and to contrast the effectiveness of two different tapered gutta-percha master cones (0.02 and 0.04). A sample of sixty single root teeth was used. Six experimental groups were formed from the three filling techniques and the two tapered master cones. Images were acquired using a confocal laser scanning microscope. In the apical root third, the penetration percentage was higher in the hybrid compared with the continuous wave technique. The results indicated a higher penetration depth of hybrid compared with cold lateral condensation in the middle and coronal thirds, and in the apical third, a higher penetration was identified in the hybrid group compared with the continuous wave group. No significant differences in penetration were found comparing 0.02 with 0.04 taper gutta-percha groups. The coronal cross-sections presented a higher penetration than the apical third sections. In conclusion, the hybrid technique a had higher maximum sealer penetration than the continuous wave in the apical third, and the coronal third hybrid and continuous wave had a higher penetration than cold lateral condensation.

## 1. Introduction

Successful root canal treatment requires an impermeable tridimensional seal along the root canal’s length, thereby ensuring the healing of periapical tissues and preventing intracanal recontamination from coronal leakage [1]. The techniques for root canal filling have improved; however, there are still challenges, such as inadequately prepared areas affecting the adaptation of obturation material [2], apical extrusion of gutta-percha and sealer, difficulty in performing the technique, the lack of hermetic sealing leading to favorable environments for bacterial colonization, and the challenge of addressing obturations in the face of different anatomical variations within endodontic spaces, ranging from oval and curved canals to lateral canals, isthmuses, and other issues, resulting in obturation failure.

The cold lateral condensation technique remains the most accepted approach for root canal filling, being a reliable technique that may be used in most cases [3]. An important advantage of cold lateral condensation is the controlled placement of the gutta-percha in the canal [4]. Additionally, it is frequently used for comparison with other compaction techniques [5]. However, cold lateral condensation has some limitations, such as potential deficiencies in gutta-percha mass homogeneity, partial filling in certain hard-to-reach areas of the root canal system, and, in the case of fragile teeth, there is a risk of root fractures [6,7,8].

Techniques based on gutta-percha heating were introduced to improve the three-dimensional filling of root canal systems. Schilder [9] developed a technique using heat and vertical condensation. This was modified, and the continuous wave condensation technique was introduced [10]. Root canal obturation with injected thermoplasticized gutta-percha was presented by Yee et al. [11] Subsequently, Tagger [12] developed a hybrid technique combining mechanical and thermal obturation with cold lateral condensation.

Thermoplastic methods offer the benefit of achieving good adaptation to the root canal walls and require less treatment time when compared to cold lateral condensation. Nevertheless, there is a possibility of gutta-percha undergoing physicochemical changes with the use of this technique. Thermomechanical compaction has demonstrated commendable adaptation, especially within the middle and coronal thirds of the root. Although there is limited supporting evidence regarding the effectiveness of a hybrid approach combining cold lateral condensation in the apical third and thermoplastic techniques in the middle and coronal thirds of the root canal, it has the potential to harness the advantages of both techniques. Yet, the assessment of filling quality in hybrid condensation techniques has yielded inconclusive findings [13,14].

The introduction of the nickel-titanium (Ni-Ti) rotary instruments in root canal preparation can improve the results and reduce the time required during the canal’s instrumentation. Rotatory instruments may help maintain the root canals’ original curvature, shape, and patency [15,16]. To match up with the various tapers of Ni-Ti instruments, firms have produced gutta-percha with a large taper (0.04 and 0.06). Gordon [17] found that higher taper gutta-percha points had better adaptation in a study of filling efficacy and reduced the time required for filling the canal compared with the standard tapered cones, using the cold lateral condensation. Nevertheless, some studies found different results [18,19].

A high percentage of endodontic sealer penetration may be an indirect indicator of potential resistance to microbial and fluid filtration between the canalicular system and the periapex, favoring three-dimensional obturations. Several factors may influence sealer penetration, including the effectiveness of the removal of the smear layer, [20] the anatomy of the root canal system, [21,22] the obturation techniques, [23] the physical and chemical properties of the sealer, [24] and the gutta-percha characteristics [17]. Confocal laser scanning microscopy (CLSM) is considered an appropriate technique for evaluating the amount of sealer that has penetrated the dentinal tubules [17,25].

The root filling efficacy of various compaction techniques employing gutta-percha points has yielded inconclusive findings, particularly in the context of hybrid techniques and the influence of different tapered gutta-percha master cones. This study postulates two key hypotheses. Firstly, it is hypothesized that the hybrid compaction technique will yield superior results in terms of root canal sealing when compared to the cold lateral compaction and continuous wave techniques. Secondly, the second hypothesis posits that the utilization of more tapered gutta-percha master cones (0.04) will result in improved sealing proficiency compared to their less tapered counterparts (0.02), leading to greater sealant penetration within the root canal dental tubules.

Accordingly, this study aims to compare the maximum sealer penetration depth and the percentage of sealer penetration into dentinal tubules using three condensation techniques: cold lateral condensation, continuous wave, and a hybrid, while employing two different tapered gutta-percha master cones (0.02 and 0.04).

## 2. Materials and Methods

The Ethics Committee of the Egas Moniz University Instituteo (CIIEM), Portugal, approved the study protocol (CEPI/0893). The sample size was calculated for the percent of sealer penetration using cold lateral condensation as a reference technique, and information from a confocal study [26], with a mean of 73 and a standard deviation of 8.7; we assumed that the experimental technique (hybrid) had a higher standard deviation (sd 14) than the reference technique. The effect size established was 15 percent points. The type I error alpha was set at 0.05 and the power (1 − β) was 0.80. The sample size obtained was n = 10. The STATA V17 (StataCorp LLC, College Station, TX, USA) program used a sample size calculation.

The root specimens for laboratory tests were obtained from Egas Moniz Instituto Universitrio CIIEM. Sixty extracted fully developed human teeth with single, straight canals were selected and radiographically examined (Kodak 2100; Kodak Dental Systems, Atlanta, GA, USA) with a Trophy RVG Ultimate 6100 digital sensor (Kodak Dental Systems, Atlanta, GA, USA) from facial and proximal views to ensure the presence of a single canal and to evaluate the root canal morphology. The study applied inclusion criteria, which required teeth to be erupted with fully developed apices and straight root canals. Exclusion criteria included teeth with visible root cracks, fractures, calcified root canals, or dental caries affecting the root portion of the tooth. Furthermore, teeth with dental development anomalies were also excluded. The teeth were stored in a 0.2% sodium azide solution.

### 2.1. Root Canal Preparation and Filling

The root length was standardized as 12 mm from the apex and cut with a 910P diamond disc (Drewdel Zweilinf, Berlin, Germany). The roots were debrided with ultrasonic scalers and washed with distilled water. Subsequently, they were immersed for 15 min at room temperature in a 6% NaOCl solution to remove the remaining organic debris, following Gharib protocol [27].

The working length of each canal was established by measuring the penetration of a 10 K-file (Flexofile; Dentsply Maillefer, Ballaigues, Switzerland) until it reached the apical foramen and then subtracting 0.5 mm. The canal spaces were cleaned and mechanically shaped with Mtwo rotary system files (VDW, Munich, Germany) in conjunction with SlickGel ES (SybronEndo Company, Orange, CA, USA) and 1.8 mL 5.25% sodium hypochlorite (NaOCl) between each file size. A handpiece was used with an electric engine (X-Smart; Dentsply Maillefer, Ballaigues, Switzerland) at 280 rpm. The manufacturer’s recommendations were followed during the root canal preparation. All the canals were enlarged to an ISO size 35, 0.04 taper, to working length.

A dentist instrumented all the teeth. Apical patency was maintained throughout the instrumentation using a #10 K-file (Flexofile; Dentsply Maillefer, Ballaigues, Switzerland). After preparation, the canals were irrigated for 3 min with 17% ethylenediaminetetraacetic acid (EDTA) to remove the smear layer, followed by 5 mL NaOCl and final irrigation with 10 mL of distilled water to avoid the prolonged effect of the EDTA and NaOCl solutions. The canals were subsequently dried with paper points. AH plus (DeTrey Dentsply, Konstanz, Germany) was used as a sealer in all the cases. This sealer was labelled with rhodamine B (0.1%) (Sigma-Aldrich, St. Louis, MO, USA) to allow analysis under confocal laser scanning microscopy (CLSM). The sealer was applied using a #35 Lentulo Spiral (Zipperer-VDW, Munich, Germany).

### 2.2. Root Canal Preparation and Filling Technique

Sixty roots were randomly divided into six groups of 10 teeth each. According to the condensation technique and the taper of the gutta-percha master cone, we formed the following groups:

Group 1 (cold lateral condensation 0.02). Ten root canals were filled with 0.02 tapered gutta-percha (VDW, Munich, Germany) using the cold lateral condensation technique. A gutta-percha cone of 0.02 taper was fitted into the root canal at the working length, making firm pressure in an apical direction. A finger spreader (Dentsply Maillefer, Ballaigues, Switzerland) number 20 was introduced at 1 mm short of the working length to laterally compact the gutta-percha to create space and be able to insert auxiliary 0.02 taper gutta-percha cones. This process was repeated until the digital spreader only entered the coronal third of the root canal. A hot metal instrument was used for trimming excess coronary gutta-percha and was subsequently compacted vertically with a Buchanan instrument.

Group 2 (cold lateral condensation 0.04). Ten roots were filled with gutta-percha with a 0.04 taper, using the cold lateral condensation as described in Group 1.

Group 3 (continuous wave 0.02). Ten root canals were sealed with gutta-percha of standardized 0.02 taper using a continuous vertical wave condensation technique with thermoplastic injection.

After inserting a standard gutta-percha point adapted to 1 mm short of the working length, the corresponding tip of the Elements Obturation Unit (EOU) sealing system was heated to 200 °C, and a depth of 3 mm above the canal’s length was used. Once the apical third was sealed, the other two-thirds of the root canal was sealed using the thermoplastic gutta-percha injection technique, elements obturation unit EOU system (SybronEndo Company, Glendora, CA, USA) by applying manual vertical condensation, and the corresponding Buchanan Pluggers instrument (Sybron Endodontics, Orange, CA, USA).

Group 4 (continuous wave 0.04). Ten roots were filled with gutta-percha with 0.04 taper, using continuous wave as described in Group 3.

Group 5 (hybrid 0.02). Ten root canals were sealed with a gutta-percha 0.02 taper using a hybrid filling technique. The cold lateral condensation and thermoplastic filling condensation techniques were combined. After filling the root canal using the cold lateral condensation as described for Group I, a hot instrument was used to remove the gutta-percha from the middle and coronal thirds. Subsequently, vertical pressure was applied using the corresponding Buchanan Pluggers (Sybron Endodontics, Orange, CA, USA) within 5 to 7 mm of working lengths to improve the gutta-percha adaptation. The coronal and middle third portions of the root canal were sealed with thermoplastic gutta-percha injected by the corresponding terminal of the elements gutta-percha cartridges (SybronEndo, Glendora, CA, USA) using the elements obturation unit (SybronEndo Company, Glendora, CA, USA). Subsequently, gutta-percha was vertically compacted with a Buchanan Pluggers instrument (Sybron Endodontics, Orange, CA, USA).

Group 6 (hybrid 0.04). Ten roots were filled with gutta-percha with 0.04 taper, using the hybrid technique described in Group 5.

**Figure 1:**
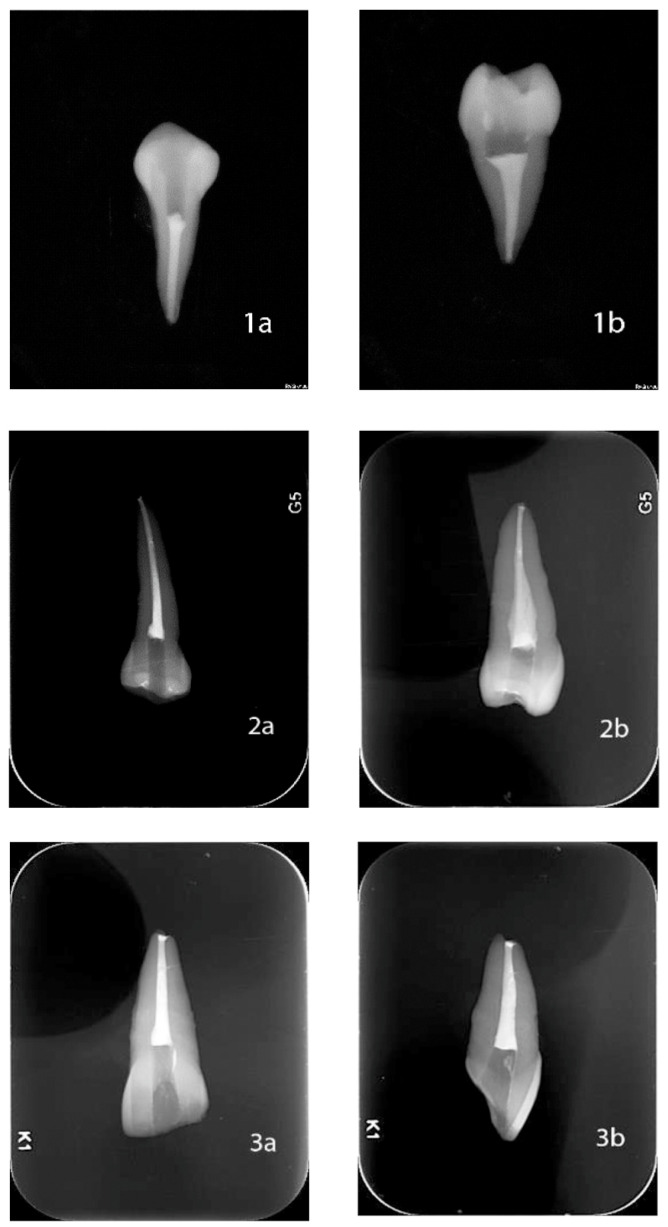
Illustrates radiographic images of teeth filled with each of the three compaction techniques evaluated. Cold lateral condensation ((**1a**) vestibule-lingual and (**1b**) mesiodistal radiograph); continuous wave ((**2a**) vestibule-lingual and (**2b**) mesiodistal radiograph) and hybrid technique ((**3a**) vestibule-lingual and (**3b**) mesiodistal radiograph).

In all the filled root canals, vestibule-lingual and mesiodistal radiographs were taken of each root to verify its correct condensation. Facial and proximal views of digital radiographs of all teeth roots were taken to verify the canal obturations. Figure 1 illustrates radiographic images depicting root canals filled using the three techniques examined in the current study.

The teeth were placed in a Memmert BE 500 heater (Memmert, Heilbronn, Germany) and incubated at 37 °C for three days to set the sealer completely. Each root was embedded in an epofix hardener resin block (Struers, Ballerup, Denmark).

### 2.3. Sectioning and Image Analysis

One-millimeter transversal sections were made in the 3, 7, and 10 mm levels from the apex of sixty roots using a 0.3 mm Isomet saw (Buehler IsoMet, Lake Bluff, IL, USA) at 200 rpm and continuous water cooling. In this manner, three slices per root were created (coronal, middle, apical), resulting in 180 pieces. The samples were then mounted onto glass slides. Only the apically facing surface of each slice was examined. All the sections were sequentially polished with Sof-Lex discs (3M ESPE, Seefeld, Germany) using running tap water as a lubricant to smooth the surfaces.

Images of the filled areas were acquired using the epifluorescence mode of an inverted Leica TCS-SP2 confocal laser scanning microscopy (CLSM) (Leica, Mannheim, Germany). An argon-mixed gas laser was used (Ar/HeNe) as the light source. Excitation light had a wavelength maximum of 543 nm. The respective absorption and emission wavelengths for rhodamine B were 554 and 649 nm. The images were recorded at 40×, with a 1024 × 1024 pixels resolution. The images were acquired and analyzed using the Leica Confocal software Version 2.1E (Leica Microsystems, Heidelberg, Germany). All the pictures were taken by using 10 sections with a 4 μm step size.

**Figure 2:**
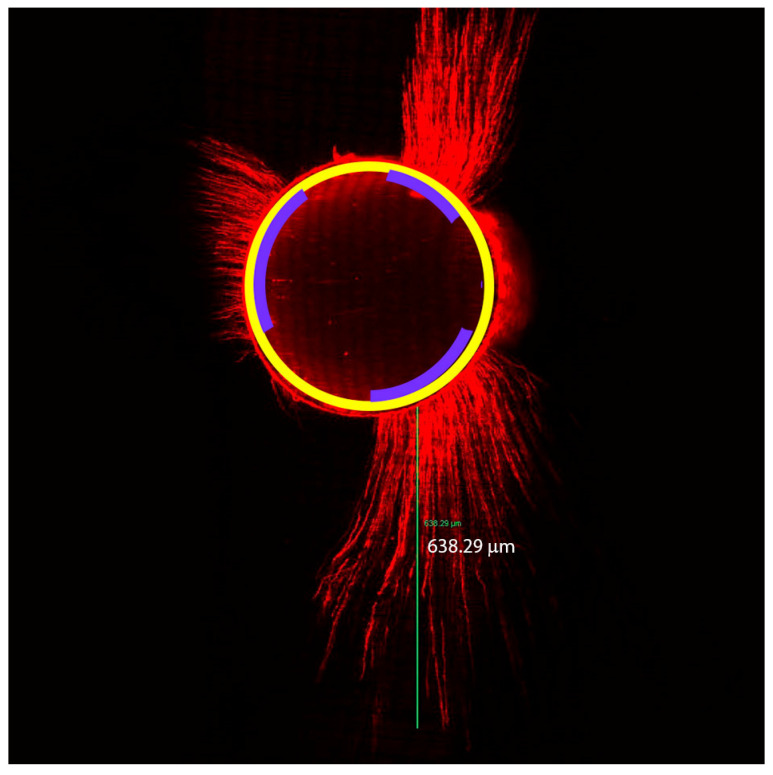
Representative confocal laser microscopic image showing the maximum depth of sealer penetration illustrated by a green line. The root canal wall with sealer penetration is illustrated using a violet curved line, and a yellow line depicts the perimeter.

The images were evaluated according to the method used by Gharib et al. [27] for measuring the depth of penetration; each image was imported into the Leica Confocal Software Version 2.1E and, using the distance tool software, the point of deepest penetration was measured from the canal wall to the area of maximum sealer penetration (Figure 2). Each image was imported into the Image J software (Rasband WS, ImageJ, 1.54; US National Institute of Health, Bethesda, MD, USA) to calculate the percentage of sealer penetration, and the root canal’s circumference wall was measured. Next, the areas along the canal walls in which the sealer penetrated dentinal tubules (sealer tags) were outlined and measured using the same method. The percentage of the canal walls where the sealer had penetrated was calculated by dividing the canal circumference’s outline by the entire circumference (Figure 2).

### 2.4. Statistical Analysis

The average percentage of penetration and the maximum depth of sealer penetration were calculated in the three root levels analysed: 3, 7, and 10 mm. These indicators were compared among the three condensation techniques used in the levels of the root canal examined using mixed linear regression models, considering that the observations were nested within teeth. Models with and without interaction terms were tested (interactions between the root level and the compaction technique, and between the technique and the gutta-percha taper master cones). Unstandardized β coefficients, and 95% confidence intervals (95% CI) were obtained. Akaike’s information criterion was used for the model selection. After fitting the models and obtaining estimates for coefficients (βo, β_1_,…, β_k_) linear combinations of these estimators were calculated in order to obtain the interaction terms effects as well as their 95% confidence intervals and *p*-values. The statistical significance value was set at α = 0.05. Data analysis was performed using Stata V15 (StataCorp LLC., College Station, TX, USA).

## 3. Results

The results of the percentage sealer penetration using the cold lateral condensation technique presented a mean of 57.49 (±28.9), for continuous wave, 56.8 (±33.1), and, for the hybrid technique, 64.5 (±29.8). Table 1 presents the mean sealer penetration by technique and gutta-percha taper across the root level. In the coronal sections, the lowest percentage of penetration corresponded to the cold lateral condensation technique (74.3%) and values above 80% were observed in the continuous wave and hybrid techniques. In the middle section, a similar pattern was observed, and in the apical third, continuous wave had the lowest percentage of penetration.

**Figure 3:**
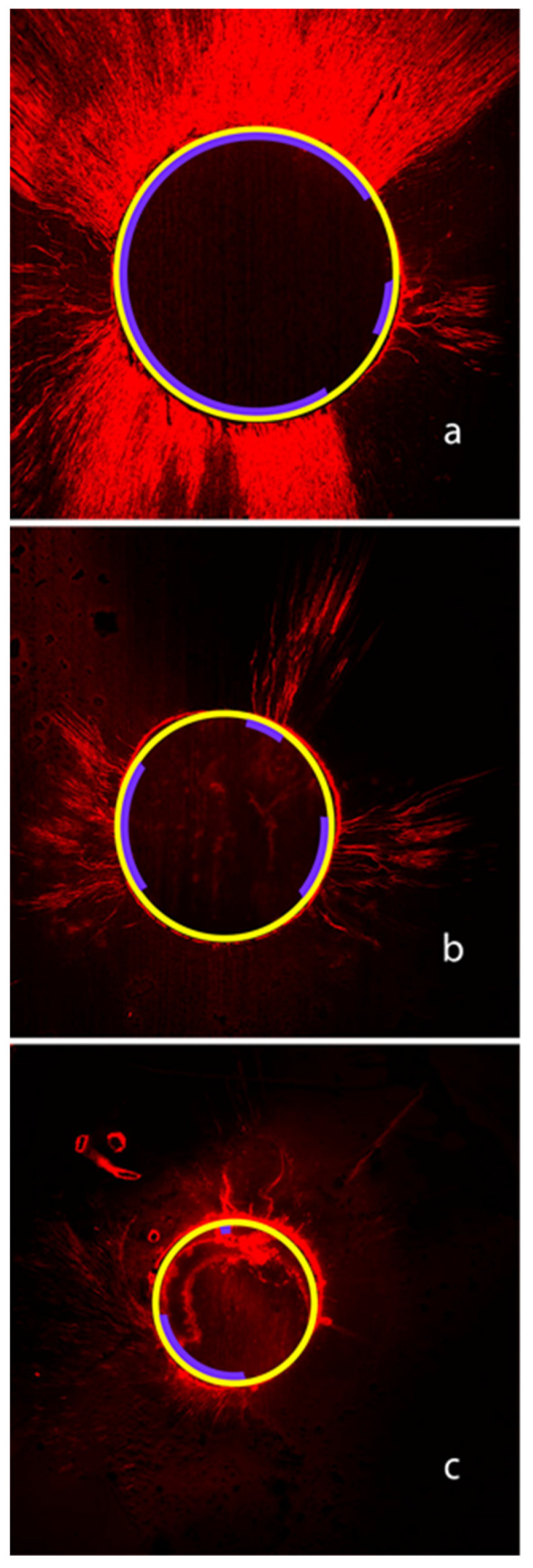
Representative confocal laser microscopic image of root sealer penetration. (**a**) corresponds to the coronal third, (**b**) depicts the sealer penetration in the middle third. And (**c**) depicts sealer penetration in the apical third, using the hybrid technique.

None of the groups studied achieved a full adaptation of sealer material on the dentin walls. Figure 3a–c illustrates the root sealer penetration into the coronal (Figure 3a), middle (Figure 3b), and apical (Figure 3c) sections, using the hybrid technique. The higher depth of sealer penetration was observed in the coronal third.

The results of the maximum penetration of sealer using the cold lateral condensation technique showed a mean of 918.2 (±383.2), for continuous wave, 960.1 (±623.4), and for hybrid, 1144.2 (±571.1). Table 2 presents the results of the maximum penetration depth of condensation techniques by root level and taper of the gutta-percha master cones. In the coronal section, the hybrid technique presented a high penetration mean (1401.6 μm), similarly, it was high in the middle section (1423.4 μm). In the coronal and middle root thirds, the lowest penetration depth was observed in the cold lateral condensation groups.

Table 3 presents the results of the mixed linear regression models fitted for percentage of penetration as the outcome variable, using as predictors the root level, technique, and gutta-percha taper size master cone (model 1 without interaction terms). In this model, the taper size was not significant (*p* = 0.803).

The root level was significant (*p* < 0.001); a higher percentage of sealer penetration was observed in the coronal compared with the middle and apical root thirds (Table 3).

A significant interaction was found between root level and technique. At the apical level, a higher percentage of sealant penetration was observed in the hybrid compared with the continuous wave technique (20.36 (IC95% 6.06, 34.65) *p* = 0.005).

**Figure 4:**
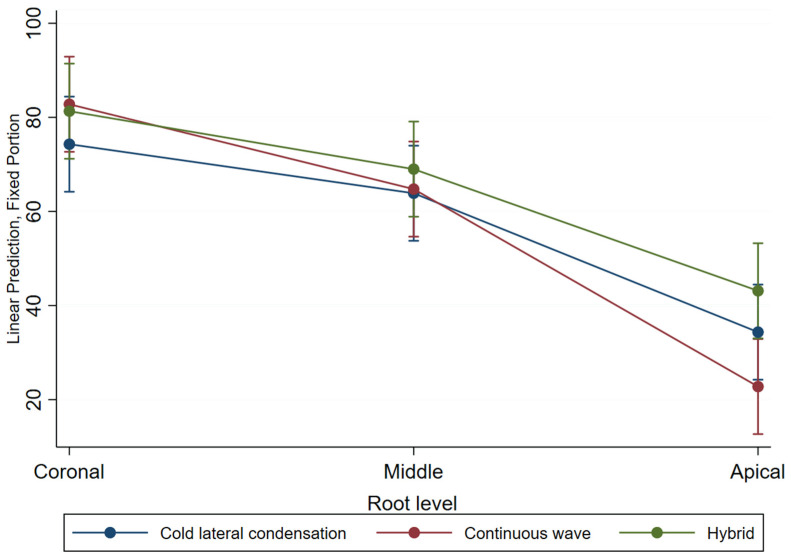
The plot of the adjusted predictors of the percentage of sealer penetration by compaction technique across root levels. Vertical bars represent 95% confidence intervals for the mean estimates.

To study the impact of possible interaction between condensation techniques and the root level on the sealer penetration, the corresponding interaction terms were included in the model (Table 3, model 2). A significant difference was observed in the apical third, indicating a higher percentage of sealant penetration in the hybrid compared with the continuous wave technique (−20.1, 95% CI (−35.6, −4.5), *p* = 0.011). Figure 4 depicts the percentage of penetration in each technique across root levels. A lack of parallelism is observed, indicating the interaction between the compaction technique and the root level. The predicted values appear to be close at the coronal and middle root levels; however, in the apical section differences between techniques were observed the continuous wave had the lowest percentage penetration results (*p* < 0.05).

Table 4, Model 1 presents the results of the mixed linear regression model fitted for maximum penetration depth, including as predictors the root level technique, and gutta-percha taper size master cone (*p* = 0.770), (Model 1). The taper size was not significant in the model, while the root level and technique was significant (*p* < 0.001), similarly to the findings of percentage penetration.

In Table 4, Model 2 presents the results of the mixed regression model including the interaction terms between the root level and the technique; this interaction was significant. The computation of the effects indicated that in the middle third, the hybrid technique had higher penetration than cold lateral condensation (440.6 95% CI (189.10, 692.06), *p* = 0.001). Additionally, in the apical third continuous wave presented a lower penetration depth than cold lateral condensation (−320.77 95% CI (−572.25, −69.30) *p* =0.012).

**Figure 5:**
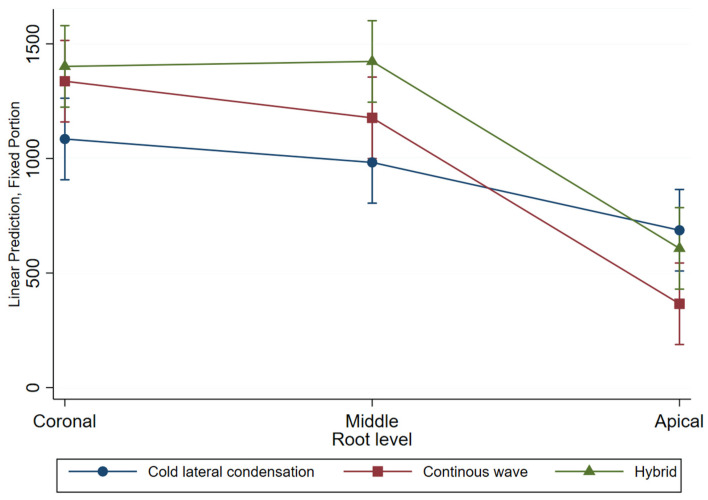
The plot of the adjusted predictors of maximum penetration depth (μm) by compaction technique across root levels. Vertical bars represent 95% confidence intervals for the mean estimates.

Figure 5 depicts the maximum depth of sealer penetration for each technique across the root level, and an interaction between the technique and the root level is observed. The predicted values of sealer penetration in the coronal third of hybrid and continuous wave are close to each other, and cold lateral condensation shows lower results, while in the apical third this relationship changes and continuous wave has the lowest penetration value.

## 4. Discussion

In this study, the penetration of sealer material into dentin tubules using three condensation techniques (cold lateral condensation, continuous-wave and hybrid) were compared. The study alternative hypothesis was that the hybrid technique would provide higher sealer penetration than cold lateral condensation. The results support this hypothesis. The study provided evidence of a significant difference between the cold lateral condensation and the hybrid technic evaluated. The hybrid method presented good results, combining the cold lateral condensation in the apical third and the continuous wave above the middle and coronal thirds.

Similarly, a study of the efficacy of filling techniques in C-1 shaped root canals found fewer voids and a higher percentage of gutta-percha using the cold lateral condensation than using the continuous wave in the apical third [21]. Accordingly, a comparison of the cold lateral condensation and the continuous wave found less sealer and more gutta-percha in the cold lateral condensation sample in the 2 mm sections. Furthermore, the continuous wave technique had more penetration than the cold lateral condensation into the 4, 6 and 8 mm sections [23].

The information obtained in the evaluation of complex root canal anatomies may support using the hybrid technique tested in the present study. Likewise, in a microcomputed tomography-based study comparing Thermafil and the cold lateral condensation, it was identified that using Thermafil voids surrounded by the filling material was mostly in the apical root third [28]. The results of these studies suggest that the middle and coronal thirds of the root canal are better filled with warm gutta-percha techniques compared with the cold lateral condensation; however, the apical third cold lateral condensation appears to have adequate results gutta-percha cones placed against the root wall during cold lateral condensation may facilitate penetration of the sealer, particularly at the apex [29]. Karatekin et al. [23] suggested the need for a modified continuous wave technique in the apical 2 mm section in C1-type canals. The hybrid technique described in the present study may be an adequate option for these cases.

Study findings on the effectiveness of hybrid or “combination” filling techniques are inconsistent. Tagger’s hybrid technique applied with the cold lateral condensation and GuttaFlow identified a larger gutta-percha-filled area in the middle and coronal thirds using this hybrid condensation technique, but there was no difference in the apical third regrading the percent of the gutta-percha area, voids and sealer area [13].

Likewise, a longitudinal study examining bacterial penetration into the root canal found no difference in the sealing ability between cold lateral condensation, Thermafill, System B, and ProTaper gutta-percha single cones after two months of observation [30]. A study using nano-computer tomography image found no difference in total obturation and calculated voids between cold lateral condensation and continuous wave [31]. Saberi et al. [32] found that a single cone technique and a hybrid (tapered cone and cold lateral compaction) technique had lower coronal leakages than the cold lateral condensation.

The inconsistencies observed in the results of various studies may be attributed to variations in experimental protocols. These variations encompass factors such as the type of teeth sections utilized, the conditions of the root canal, differences in the preparations of the root canals, the specific type of endodontic sealer selected, the treatment steps, the methodologies employed for assessing sealer penetration, and the overall experimental design, among other variables [33,34]. Clinical variations among teeth may also contribute to disparities, including distinctions in the anatomy of root canals, patient-related factors, and variations in dentin structure across different sections of teeth dentin sclerosis has been found to influence sealer penetration [35].

The apical portion of human teeth exhibits significant structural variations, such as accessory root canals, areas of resorption, pulp stones, irregular secondary dentine, cementum-like lining, and deviations in the apex from the root canal’s long axis [36]. As individuals age, dentin undergoes permanent microstructural modifications characterized by the gradual mineral infiltration of tubule lumens. This process, referred to as dental sclerosis, commences at the root apex, progresses towards the crown, and results in a decreased fracture resistance of dentin [37]. This may help elucidate the greater sealer penetration observed in the coronal region compared to the apical region in our current study.

The time elapsed between obturation and the assessment of sealer penetration, as well as environmental factors affecting sealer setting, can exert an impact on the obtained results. It should be recognized that sealer penetration may undergo changes as the sealer sets and interacts with dentin over time [38,39]. Addressing these factors and maintaining standardized methods can help reduce discrepancies in the results of sealer penetration studies conducted using different compaction techniques in endodontics.

The variations in study results can also be influenced by the statistical data analysis methods. In this study, mixed linear models were employed, allowing for the nesting of root sections within the teeth from which they were obtained. This nesting was considered based on a comparison between nested and non-nested models. Furthermore, the analysis of interactions between root level and condensation techniques provided insights into the differences between these techniques. The mixed linear regression model indicated the presence of an interaction between the technique and the root level. Specifically, a higher maximum penetration depth was observed in the apical root level with the hybrid technique compared to lateral condensation.

In the present study, AH Plus endodontic sealer was chosen because it has good penetration, and adequate flow and adaption ability in the root canal system, it has been considered as a gold standard [40,41,42]. The teeth were evaluated using confocal laser scanning microscopy with the incorporation of a fluorescent dye (rhodamine B). This technique displays the sealing material’s adaptation through the dentinal tubules in the specimen cross-sections [27]. In the present study, using overexposure images by the confocal laser scanning microscopy program, it was possible to obtain the offset of the dentinal tubules’ undulating path. This process provides detailed information and reduces the risk of cracking artefacts since the sample does not require drying.

The rhodamine B concentration (0.1%) was considered adequate for identifying the sealer in dentinal tubules. This fluorophore does not harm the sealer and behaves satisfactorily when it is used in combination with an epoxy resin-based material, such as the AH-plus used in the present study [13,15]. Nevertheless, the results of a study conducted by Donnermeyer et al. [43] indicated that Rhodamine B may leach into dentine beyond the sealant penetration and may be inadequate to assess sealing capacity.

However, a study by Eğemen and Belli [44] found adequate results utilizing this fluorescent dye. They investigated the effect on dentin tubules penetration of resin-based (AH-plus) and calcium silicate-based sealers. The authors conducted a pilot study to select the dyes to be used for the evaluation of sealant penetration, rhodamine (Rhod-2) and Fluo-3 were tested. The results indicated that these two dyes were appropriated to identify the sealant tubules’ penetration for the original obturation and for root canal retreatment [44].

In the present study, there were no substantial variations observed in terms of depth of penetration or the percentage of sealer penetration when comparing 0.02 tapered master cones to their 0.04 tapered counterparts. Consequently, the study did not corroborate the hypothesis proposing enhanced results with less tapered cones. Hembrough et al. [45] consistently did not detect a significant difference when measuring the percentage surface of the root canal taken up by gutta-percha, comparing 0.02 and 0.06 taper cones.

Other studies had similar results using the cold lateral condensation [17,18]. Moreover, in a study applying area-metric analysis comparing 0.02 and 0.04 taper gutta-percha, no statistically significant difference was found in the sealer-filling canal area with the cold lateral condensation or single cone techniques [19]. When assessing curved root canals, a study found no significant difference in apical leakage between 0.06 and 0.02 gutta-percha taper cones. However, canals filled with a 0.02 master cone had a lower average leakage [46]. In a study by Schäfer et al. [47], it was observed that matching the preparation with single cones in the apical section led to a higher percentage of gutta-percha-filled areas when using constant taper gutta-percha compared to variable tapered single-cone gutta-percha. The inconsistency in the results of studies with larger taper compared with standard taper gutta-percha cones may be primarily due to the instruments selected for the root canals preparation, the microleakage assessment method and the evaluation of the sealer penetration technique.

The study found that none of the compaction techniques that were evaluated achieved complete adaptation to dentinal walls, a result in line with earlier research [48]. Even though root canal treatment has a high success rate, the search for the ideal compaction technique is an ongoing endeavor.

Irrespective of the technique applied and the gutta-percha taper selected, higher penetration depth and percentage of sealer penetration were observed in the coronal third followed by the middle third and, last, the apical third. Similar results have been reported by Akcay et al. [25] when comparing different types of sealers. Moreover, these results are consistent with several studies’ findings using the confocal laser scanning microscopy technique [26,27]. In an evaluation of bonding to the root canal, significantly greater coronal dentin permeability was observed than the apical third and was influential in root bonding [49]. Higher sealer penetration in the coronal third may be explained by the reduced accumulation of the smear layer or its easier removal in this third than the middle and the apical thirds. Furthermore, the dentinal tubules’ setup in the apical region is irregular and more prone to obliteration, presenting dentin sclerosis, which hinders the penetration of the sealer cement [50]. The consistency of these results suggests that the most influential factor of this observation is the root canal anatomy. This aspect makes us reflect on the importance of performing several observations along the root canal when studying root canal compaction techniques, including the middle and coronary root levels, avoiding the omission of important information.

A limitation of the current study pertains to the exclusive inclusion of single-rooted teeth with straight canals. Root canals exhibiting diverse anatomical variations may yield distinct outcomes. Another limitation was the absence of a control group. However, all the technique steps were carefully followed and samples without root canal compaction were tested before-hand, including the experimental groups, to confirm the fluorescent dye’s penetration. Those samples covered with nail polish exhibited no dye penetration. In future studies, it is essential to explore the use of fluorescent dyes specifically designed for dentin tubule penetration. Additionally, a limitation of the fluorescent confocal laser scanning microscopy method is that the number of measurements performed in each root canal could impact the percentage and the depth of penetration the percentage and depth of penetration results. To address this limitation, the ImageJ program was utilized to measure the area and collect pixel value statistics, enabling the evaluation of both depth and the percent sealer penetration, as previously demonstrated in other studies [27].

In the realm of future research, innovative imaging methods, notably microcomputed tomography (Micro-CT), may be integrated to unlock valuable insights into the dynamic interactions between sealers and dentin. Micro-CT’s three-dimensional capabilities empower a comprehensive assessment of sealer penetration, revealing its volumetric characteristics and spatial distribution within dentin. This technique can effectively complement the information offered by confocal laser scanning microscopy (CLSM), a potent imaging tool recognized for its high-magnification examination of dentin surfaces and sealer distribution patterns [51]. In addition, the integration of Micro-CT or 2D printed replicas presents an opportunity to enhance the precision of tooth comparison in endodontic procedures, effectively mitigating potential anatomical discrepancies. Furthermore, the necessity for long-term scrutiny of sealer penetration becomes apparent, as the longevity of felling materials may become a significant factor [51].

In a systematic review and meta-analysis conducted by Mekhdieva et al. [52] the objective was to evaluate the effectiveness of bioceramic endodontic sealers in reducing postoperative pain when compared to traditional filling techniques. Their analysis revealed that patients who received treatment with bioceramic sealers, whether in a single visit or across multiple visits, reported experiencing lower levels of postoperative pain [52]. Future research can investigate the effects of innovative biocompatible sealing materials, such as bioceramic sealers, on hybrid compaction techniques like the one introduced in our present study.

This study adds to the knowledge on sealer penetration in favor of using hybrid techniques. The combination of cold lateral condensation and continuous wave has the advantage of using a classic technic, which has a relative low cost and is taught in most of dental schools, with continuous wave that requires lower obturation time and allows good adaption to the root canal complexities [6].

This study significantly contributes to the existing body of knowledge pertaining to sealer penetration, underscoring the merits of employing hybrid techniques in endodontic treatment. The combination of traditional cold lateral condensation alongside the modern continuous wave method offers a distinctive advantage. It combines a well-established, cost-effective classic technique that is widely integrated into dental education curricula with the efficiency of the continuous wave approach, resulting in reduced obturation time and enhanced adaptability to the intricate configurations of the root canal system.

## 5. Conclusions

In conclusion, the depth of the penetration of root canal sealers into the dentinal tubules and the percentage of sealer penetration into the root canal walls were significantly influenced by the root canal level, with a lower penetration into the root canal apical third. Teeth filled with gutta-percha master point taper 0.04 had a similar maximum sealer penetration and percentage sealant penetration to conventional 0.02 taper points. The ability to obtain good sealer penetration was different between the techniques across root levels. The hybrid technique evaluated, combining the cold lateral condensation in the apical root third and continuous wave above the apical third, presented a higher maximum sealer penetration into the apical third than the continuous wave technique, and higher penetration than the cold lateral condensation was observed into the middle and coronal thirds. The evaluated hybrid technique may be a good alternative in root canal treatment.

## Figures and Tables

**Table 1:** Percentage of sealer penetration in μm into dentinal tubules in the coronal (10 mm), the middle (7 mm), and the apical (3 mm) thirds by groups according with technique and gutta-percha point taper size.

Groups Condensation Technique and Gutta-Percha Taper	Root Level
	Coronal (10 mm)
	Mean (±SD)
Cold Lateral Condensation	74.3 (19.1)
Cold Lateral Condensation 0.02 ^1^	75.4 (20.5)
Cold Lateral Condensation 0.04 ^2^	73.2 (18.6)
Continuous Wave	82.7 (21.0)
Continuous Wave 0.02 ^1^	74.3 (26.1)
Continuous Wave 0.04 ^2^	91.3 (9.6)
Hybrid	81.3 (20.9)
Hybrid 0.02 ^1^	79.6 (22.3)
Hybrid 0.04 ^2^	83.0 (20.4)
	Middle (7 mm)
	Mean (±SD)
Cold Lateral Condensation	63.9 (25.2)
Cold Lateral Condensation 0.02 ^1^	62.9 (24.1)
Cold Lateral Condensation 0.04 ^2^	64.8 (27.5)
Continuous Wave	64.8 (19.5)
Continuous Wave 0.02 ^1^	63.5 (23.1)
Continuous Wave 0.04 ^2^	66.0 (16.4)
Hybrid	69.0 (27.9)
Hybrid 0.02 ^1^	75.4 (22.9)
Hybrid 0.04 ^2^	62.6 (22.1)
	Apical (3 mm)
	Mean (±SD)
Cold Lateral Condensation	34.3 (26.4)
Cold Lateral Condensation 0.02 ^1^	37.5 (28.8)
Cold Lateral Condensation 0.04 ^2^	31.3 (24.9)
Continuous Wave	22.8 (23.9)
Continuous Wave 0.02 ^1^	20.1 (31.6)
Continuous Wave 0.04 ^2^	25.4 (14.0)
Hybrid	43.1 (31.5)
Hybrid 0.02 ^1^	33.5 (28.2)
Hybrid 0.04 ^2^	52.8 (33.1)

^1^ Gutta-percha master cone taper 0.02, ^2^ Gutta-percha master cone taper 0.04.

**Table 2:** Depth of sealer penetration in μm into dentinal tubules in the coronal (10 mm), the middle (7 mm), and the apical (3 mm) thirds, by groups according with condensation technique and gutta-percha taper sizes.

Group by Condensation Technique and Gutta-Percha Taper	Root Level
	Coronal (10 mm)
	Mean (±SD)
Cold Lateral Condensation	1084.9 (197.9)
Cold Lateral Condensation 0.02 ^1^	1294.5 (450.1)
Cold Lateral Condensation 0.04 ^2^	875.5 (461.5)
Continuous Wave	1337.1 (291.1)
Continuous Wave 0.02	1271.9 (678.4)
Continuous Wave 0.04	1402.8 (514.0)
Hybrid	1401.6 (477.3)
Hybrid 0.02	1170.7 (796.1)
Hybrid 0.04	1632.9 (585.8)
	Middle (7 mm)
	Mean (±SD)
Cold Lateral Condensation	982.8 (467.4)
Cold Lateral Condensation 0.02 ^1^	1187.7 (552.7)
Cold Lateral Condensation 0.04 ^2^	778.0 (449.5)
Continuous Wave	1177.3 (694.0)
Continuous Wave 0.02	1152.1 (666.0)
Continuous Wave 0.04	1202.5 (756.1)
Hybrid	1423.4 (442.8)
Hybrid 0.02	1394.7 (506.2)
Hybrid 0.04	1452.0 (394.8)
	Apical (3 mm)
	Mean (±SD)
Cold Lateral Condensation	686.8 (329.1)
Cold Lateral Condensation 0.02 ^1^	711.4 (412.9)
Cold Lateral Condensation 0.04 ^2^	662.3 (565.2)
Continuous Wave	366.0 (263.8)
Continuous Wave 0.02	244.7 (280.5)
Continuous Wave 0.04	487.4 (422.5)
Hybrid	607.7 (365.9)
Hybrid 0.02	745.5 (586.9)
Hybrid 0.04	470.0 (501.1)

^1^ Gutta-percha master cone taper 0.02, ^2^ Gutta-percha master cone taper 0.04.

**Table 3:** Regression coefficients of percentage of sealer penetration and root level and type of condensation technique, and gutta-percha cone tapper (model 1), and regression coefficients of maximum penetration depth, technique and interaction between root level and technique (model 2).

**Model 1** ^1^			
**Variable**	**Coefficient**	**95% (CI)**	***p***
Root level			
Coronal	ref	-	-
Middle	−13.6	(−20.2, −7.0)	<0.001
Apical	−46.1	(−52.7, −39.5)	<0.001
Technique			
Cold lateral condensation	ref	-	-
Continuous wave	−0.8	(−11.9, 10.4)	0.895
Hybrid	7.0	(−4.2, 18.1)	0.219
Tapper			
0.02	ref	-	-
0.04	−1.2	(−10.3, 7.9)	0.803
**Model 2** ^1^			
**Variable**	**Coefficient**	**95% (CI)**	***p***
Root level			
Coronal	ref	-	-
Middle	−10.4	(−21.4, 0.6)	0.063
Apical	−40.0	(−50.9, −29.0)	<0.001
Technique			
Cold lateral condensation	ref	-	-
Continuous wave	8.5	(−5.8, 22.8)	0.244
Hybrid	7.0	(−7.3, 21.3)	0.336
Root level and technique ^2^			
Coronal × Cold lateral condensation	ref	-	-
Middle × Continuous wave	−7.6	(−23.1, 7.9)	0.337
Middle × Hybrid	−1.9	(−17.4, 13.7)	0.812
Apical × Continuous wave	−20.1	(−35.6, −4.5)	0.011
Apical × hybrid	1.8	(−13.8, 17.3)	0.823

^1^ Nested model (root level nested in teeth), Likelihood Ratio test vs. linear model: χ^2^ = 27.32 *p* < 0.001. ^2^ Interaction root level and type of technique.

**Table 4:** Regression coefficients of maximum penetration depth and root level. Type of condensation technique and gutta percha cone tapper (Model 1). Regression coefficients of maximum penetration depth, condensation technique and interaction between root level and technique (Model 2).

**Model 1** ^1^			
**Variable**	**Coefficient**	**95% (CI)**	***p***
Root level			
Coronal	ref	-	-
Middle	−102.1	(323.8, 119.6)	0.367
Apical	−398.1	(−619.9, −176.4)	<0.001
Technique			
Cold lateral condensation	ref	-	-
Continuous wave	252.2	(0.739, 503.7)	0.049
Hybrid	316.7	(65.3, 568.2)	0.014
Taper			
0.02	ref	-	-
0.04	−21.2	(−163.6, 121.2)	0.770
**Model 2** ^2^			
**Variable**	**Coefficient**	**95% (CI)**	***p***
Root level			
Coronal	ref	-	-
Middle	−102.1	(−323.8, 119.6)	0.367
Apical	−398.1	(−619.9, −176.4)	<0.001
Technique			
Cold lateral condensation	ref	-	-
Continuous wave	252.2	(0.7, 503.7)	0.049
Hybrid	316.8	(65.3, 568.2)	0.014
Root level and technique ^3^			
Coronal × Cold lateral condensation	ref	-	-
Middle × Continuous wave	−57.7	(−371.3, 255.9)	0.718
Middle × Hybrid	123.8	(−189.7, 437.4)	0.439
Apical × Continuous wave	−573.0	(−886.6, −259.4)	<0.001
Apical × Hybrid	−395.8	(−709.4, −82.3)	0.013

^1^ Nested model (root level nested in teeth). ^2^ Likelihood Ratio test vs. linear model: χ^2^ = 8.12, *p* = 0.002. ^3^ Interactions terms root level and type of technique.

## Data Availability

Data will be available from corresponding authors.
